# Top soil physical and chemical properties in Kazakhstan across a north-south gradient

**DOI:** 10.1038/sdata.2018.242

**Published:** 2018-11-13

**Authors:** Vadim Yapiyev, Charles P. Gilman, Tolganay Kabdullayeva, Akmaral Suleimenova, Aizhan Shagadatova, Azat Duisembay, Sanzhar Naizabekov, Saule Mussurova, Kamilya Sydykova, Ilyas Raimkulov, Ilyas Kabimoldayev, Ainagul Abdrakhmanova, Symbat Omarkulova, Dastan Nurmukhambetov, Aliya Kudarova, Daniyar Malgazhdar, Christian Schönbach, Vassilis Inglezakis

**Affiliations:** 1 Department of Civil and Environmental Engineering, School of Engineering, Nazarbayev University, Astana, Kazakhstan; 2 National Laboratory Astana, Nazarbayev University, Astana, Kazakhstan; 3 Department of Biology, School of Science and Technology, Nazarbayev University, Astana, Kazakhstan; 4 Environmental Science & Technology Group (ESTg), Chemical Engineering Department, School of Engineering, Nazarbayev University, Astana, Kazakhstan; 5 International Research Center for Medical Sciences, Kumamoto University, Kumamoto, Japan

**Keywords:** Carbon cycle, Carbon cycle, Agroecology

## Abstract

Kazakhstan’s soil properties have yet to be comprehensively characterized. We sampled 40 sites consisting of ten major soil types at spring (wet) and late-summer (dry) seasons. The sample locations range from semi-arid to arid with an annual mean air temperature from 1.2 to 10.7 °C and annual precipitation from less than 200 to around 400 mm. Overall topsoil total (STC), organic (SOC), and inorganic (SIC) carbon did not change significantly between spring and late summer. STC and SOC show a wave like pattern from north to south with two maxima in northern and southern Kazakhstan and one minimum in central Kazakhstan. With a few exceptions SIC content at northern sites is generally low, whereas at Lake Balkhash SIC can exceed 75% of STC. Independent of the seasons, SOC significantly differed among soil types. Total nitrogen content distribution among our sampling sites followed a similar pattern as SOC with significant differences between soil types occurring in northern, central and southern Kazakhstan.

## Background & Summary

Kazakhstan is the largest land-locked country in the world. Its massive land area of 2.725 × 10^6^ km^2^ represents a key reservoir for soil organic carbon that is thought to play an important role in global climate-carbon modelling^1,2^. Yet SOC data pertaining to Central Asia^3^ and particularly Kazakhstan^4^ are scarce, resulting in considerable ignorance of the extent to which forests and steppe soils in the north and east, and arid areas in the south and west, contribute to sequestering atmospheric carbon or yielding carbon into the atmosphere. Modelling approaches, such as the arid ecosystem model (AEM), yielded high soil organic carbon densities of up to 34 Pg for top soils of 1 m depth and 12–14 Pg for 30 cm deep top soils in temperate deserts of Central Asia^5^. Sub-regional SOC studies utilizing satellite imagery of farm land in northern Kazakhstan and temporal carbon variations lacked predictive power^6^. Normalized difference vegetation index (NDVI) values that reflect vegetative sensitivity to climate change were found to be high in northern Kazakhstan, and in central Kazakhstan, and NDVI changes were positively correlated with the annual temperature^7^. Net ecosystem CO_2_ exchange (NEE) studies of two sites representing alkaline desert soils near Lake Balkhash and the Aral Sea revealed CO_2_ flux dependence on moisture, pH and light^8^. The authors reported net CO_2_ release nocturnally and on cloudy days with precipitation, whereas on sunny and dry days, CO_2_ was taken up. Overall, the current picture of carbon cycling in Kazakhstan is supported by only a few sites and little data. In addition numerous SOC centric studies^9–11^ make extrapolations on regional and global carbon cycles without taking into account soil inorganic carbon (SIC), a likely sink of secondary carbonates, particularly in arid areas. According to Lal *et al.*^12^, SIC accumulation is high in arid and semiarid regions, for example, grass lands which are thought to harbour one fifth of global soil carbon stocks^13^. SIC accumulation in top soils (15 cm) was shown to be largely dependent on soil pH^14^.

More data and improved coverage of fundamental soil properties including pH, moisture content, particle size, and cation and anion composition that influence microbial activity, and thus the rate of SOC decomposition, are needed to synthesize better agro-economic and ecological strategies, especially regarding climate change predictions. Towards this end we assessed the current soil properties of Kazakhstan by sampling top soil (15 cm) at 40 sites between Petropavlovsk (north) and Taraz (south) during wet and dry seasons and determined the physical and chemical compositions, related vegetation, land cover and climate properties.

## Methods

### Study sites

**Figure 1:**
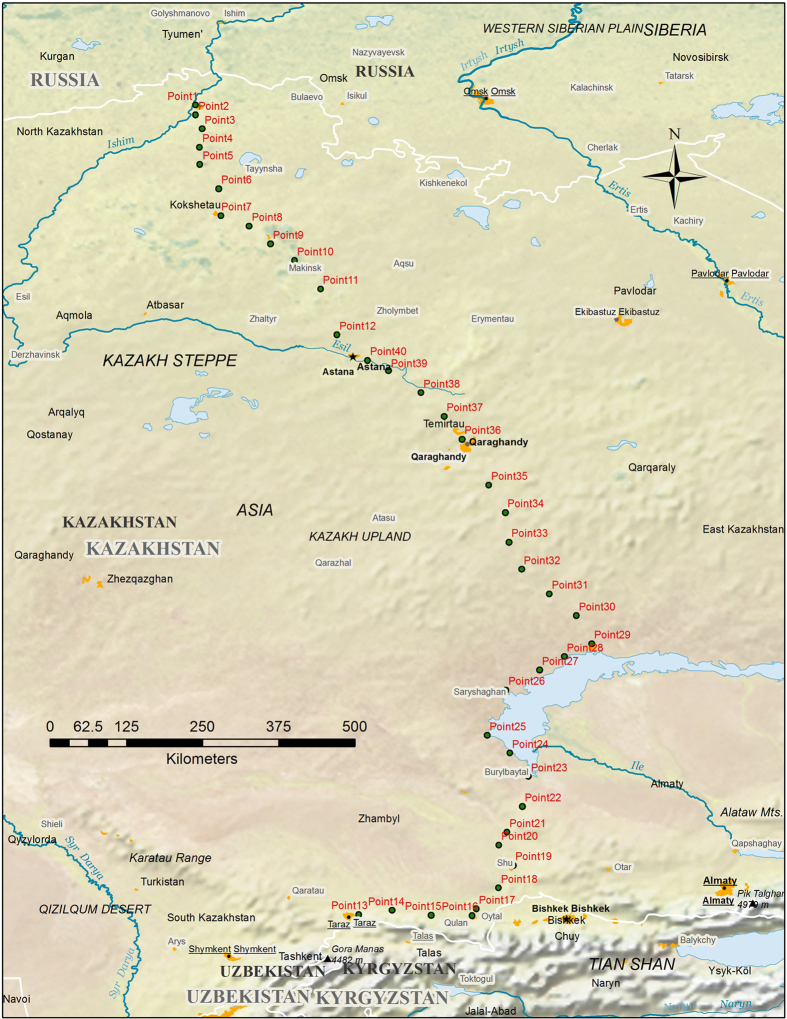
Locations of soil sampling sites. Made with Natural Earth (http://www.naturalearthdata.com).

The study has 40 sampling sites that were located a minimum of 50 m from the nearest road. Sample sites were approximately 50 km apart with flat topographic conditions (toeslope) (Fig. 1). No permits were required for the sampling site locations and the sites did not harbour endangered or protected species. Geographical coordinates (WGS-84) were recorded using a Garmin T650 hand-held global positioning system. The coordinates and geographical annotations are shown in Table 1 (available online only). Sampling was conducted in 2015: one in the “wet” season after the snowmelt (May), and the other in the “dry” season at the end of the growing season (September).

### Field Methods

#### Sampling site documentation

Pictures of the landscape and vegetation were taken at each location (see Supplement 1). The soil sampling procedure was divided into physical properties sampling, chemical sampling, and biome sampling. Soil sample preparation conditions for chemical and physical analyses (including depth of cores, particle size and milling) were chosen to be harmonized with future analyses of soil microbiomes^15,16^. Samples for biome (not subject of this paper), physical and chemical analysis parts (see Laboratory Methods) were transported on ice and transferred to 4 °C refrigerators for extended storage.

#### Samples for physical analyses

Samples for gravimetric moisture determination were obtained by digging a 10 cm diameter hole with a spade. The spade was rinsed well with distilled water between samples. Approximately 1 kg of soil was excavated, mixed and sealed in a 1 L plastic bag. For bulk density determination, a 50 ml conical tube (Corning Inc.) was filled with undisturbed soil derived from 15 cm deep cores and weighed on a portable balance (Maxx-412, Denver Instrument). At five sample sites additional samples for bulk density measurements were obtained to verify the cone method against the traditional soil ring method.

#### Samples for chemical analyses

After removing the litter layer if present, (approximately top 2 cm) soil cores of 1.5 cm in diameter were taken to depth of approximately 15 cm. The cores were transferred into 50 ml falcon tubes that were sealed with Parafilm M (Bemis Company, Inc.).

### Laboratory Methods

#### Soil moisture and bulk density

Soil moisture was calculated gravimetrically as wet and dry soil weight ratios. Briefly, triplicates of 10 g of soil were placed on aluminium paper, weighed and transferred to a 105 °C ventilated oven (Heraeus LUT 6050, Therma Fisher Scientific Inc.). After 24 h, samples were removed, immediately weighed and the dry mass recorded. Volumetric soil moisture content was calculated based on oven-dry bulk densities assuming 15 cm soil depth and by subtracting the mass of evaporated water from the wet bulk density. The volumetric soil moisture and calculated carbon stock values represent averages from two oven-dried bulk densities values obtained from two independent samples.

#### Soil milling for pH, conductivity and elemental analysis

Ten grams of dried and sieved soil of smaller than 150 μm particle size^15^ was transferred into the jar of a vibrating ball mill (MM 400, Retsch GMBH) for 2 min milling at 30 Hz. The milled sample was collected in a 15 ml Falcon tube. The vibrating ball mill processing reduces the sample particle size to approximately 1 μm.

#### Soil pH and conductivity

Soil pH and electrical conductivity were measured in triplicate in aqueous solution suspension (supernatant) with a 1: 2.5 (soil : water) ratio according the protocol of Pansu and Gautheyrou (2006) using a 8107UWMD Ross Ultra pH/ATC triode (Thermo Fisher Scientific, USA) and Orion 013010MD conductivity cell (Thermo Fisher Scientific, USA) electrodes.

#### Total soil carbon (SC), organic (SOC) and inorganic carbon (SIC) based on elemental analysis

**Figure 2:**
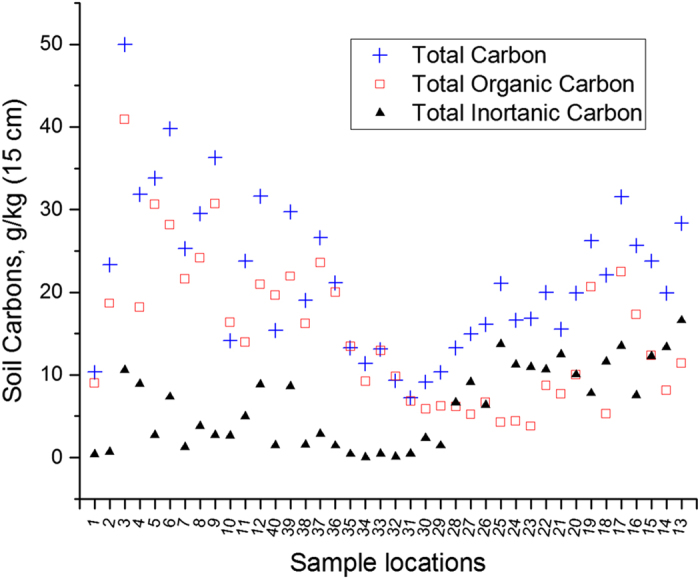
Soil carbon as total soil cabon (blue crosses), soil organic carbon (red squares), soil inorganic carbon (black triangles). See also Table 2.

Total SC and SOC were measured using a CNHS-O dry combustion elemental analyzer Multi N/Cb 3100, (Analytik Jena, Germany). For total SC 100 mg milled soil was placed in a ceramic combustion boat and combusted under pure oxygen at a flow rate of 2.8 L/min at 950 °C. The CO_2_ emitted during combustion is detected by a thermal conductivity detector. Milled soil samples for SOC estimation were pre-treated in combustion boats with 100 μl of H_3_PO_4_ (30–40%) to dissolve carbonates. Samples were dried overnight at 70 °C and subjected to combustion at 950 °C under 14 L/min oxygen (Multi N/Cb 3100, (Analytik Jena, Germany)). SIC was calculated as the difference between total SC and SOC (Fig. 2 and Table 2).

#### Loss-on-ignition (LOI) procedure for soil organic matter (SOM)

The soil samples were dried and sieved through a 2 mm sieve. We adapted the LOI method described by Emmett *et al.* (2007; Countryside Survey: Soils Report from 2007). In brief, crucibles were washed and then rinsed three times with distilled water, dried for 40 min at 105 °C in an oven (Carbolite PN 60) and then cooled to room temperature (RT) in a desiccator for 30 min. Each crucible weight was recorded (Wc) and 10.00 g of crushed soil sample (<2 mm) was weighed in the crucible, dried for four hours at 105 °C and cooled to RT in a desiccator for 30 min and the dry sample weight (Ws) recorded. Dried samples were loaded into a muffle furnace (Carbolite ELF 11/6B), heated to 375 °C for 16 h and allowed to cool down to 150 °C before being transferred to a desiccator for 30 min to cool to RT. The weight of the samples was recorded as Wa and LOI was calculated as (Ws − Wa)/(Ws − Wc) × 100.

#### Total nitrogen (TN) measurement by elemental analysis

**Figure 3:**
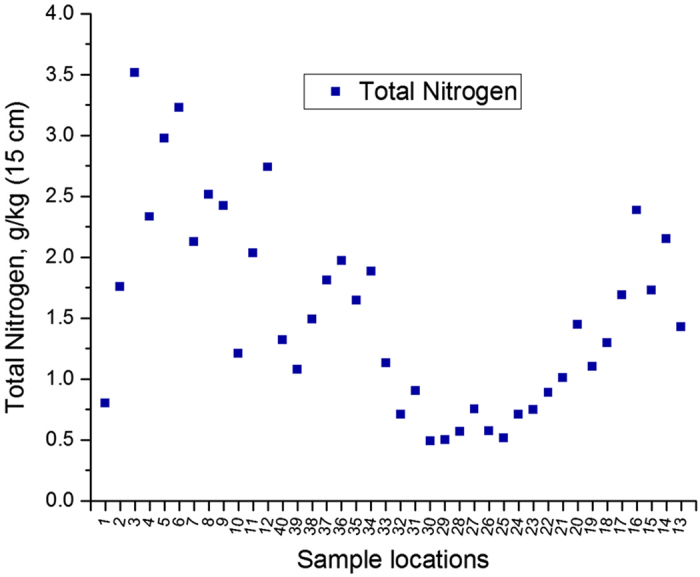
Total soil nitrogen.

The total nitrogen content of each sample was analyzed by quantitative combustion in excess oxygen using DuMaster D-480 analyzer (Büchi Labortechnik AG). L-glutamic acid was used for calibration (N-factor) of the sample measurement series. Dried and milled samples were weighed in portions of 700 mg, packed in tin foil and loaded on the sample carousel for total nitrogen measurement according to the manufacturer’s instructions (Fig. 3).

### Climate data

The Climate Research Unit high-resolution dataset (CRU TS v. 3.24.01 https://crudata.uea.ac.uk; January 26, 2017 release) contains air temperature and precipitation data ranging from 1901 until 2015 at 0.5° resolution of grid-boxes^17^ Google Earth Interface Pro was used to download the raw monthly temperature and precipitation data for the sample locations. If multiple sample locations (1–3, 4–5, 32–33, 28–29, and 19–20) mapped to one grid-box, the same temperature and precipitation data were used (e.g., sample sites 1–3 were assigned the same climate data). The climate data are presented in Table 1 (available online only).

### Carbon and nitrogen stocks calculation

**Figure 4:**
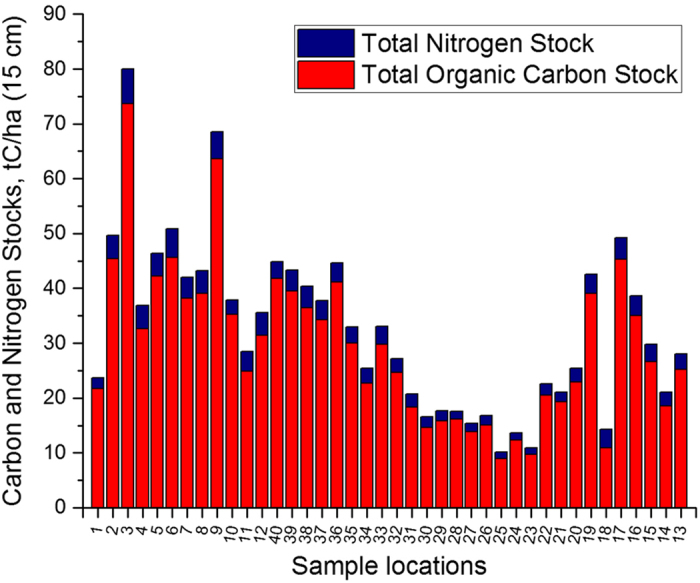
TOCand TN stocks in 15 cm of top soil.

To infer soil total organic carbon (TOC) and nitrogen (TN) stocks in tons of TOC and TN per hectare (tC/ha, tN/ha) we followed the procedure outlined by Rowell (1994)^18^, (section 3.7, p. 55). The soil layer was assumed to represent the 0–15 cm depth. We used oven dry bulk densities averages from the two samplings (May and September). For TOC and TN absolute content (g/kg or mg/g) we used mean values of averaged duplicate measurements (elemental analysis) obtained from two samplings (May and September) see Fig. 4.

## Data Records

The data were deposited at the Mendeley data repository (Data Citation 1) as Supplement 1 and 2. Supplement 1 contains a folder with photos of landscape and vegetation for each sample location. The naming convention is location number. photo number (date the sample was taken). For example, 1.1(25.05.15) corresponds to location 1 and photo 1 on May 25, 2015. For the images presented in our data records which feature identifiable human participant(s) the informed consent was obtained from the participants prior to publication of the images. Supplement 2 is a Microsoft Excel file that contains spreadsheets with data on 1) soil TC, soil total organic carbon (TOC), soil total inorganic carbon (TIC), 2) Loss on Ignition (LOI), 3) soil TN, 4) soil dry bulk density, 5) gravimetric and volumetric soil moisture 6) soil suspension and supernatant pH, 7) soil electrical conductivity, and 8) soil TOC and TN 9) annotation table (Table 1 (available online only)). Values of sample measurements from each location were reported with standard deviation (STDEV) and standard error (SE) when applicable.

## Technical Validation

**Figure 5:**
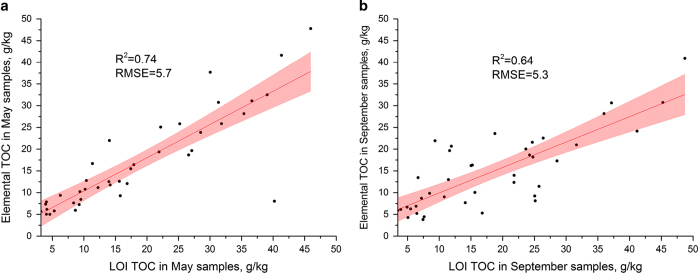
Comparison of TOC values derived from elemental analysis and LOI. (**a**) May samples. (**b**) September samples. R^2^: coefficient of determination, RMSE: root mean squared error, and red-shaded area: 95% confidence interval.

Soil carbon and nitrogen concentrations were averaged from duplicate sample preparations and validated using reference standards. If the concentrations deviated significantly the measurements were repeated. SOC results were validated independently by conducting LOI measurements (see Fig. 5). Soil organic matter (SOM) (g/kg) data obtained from LOI analyses were converted to TOC (g/kg) by multiplying the values with the coefficient 0.58^19^.

## Additional information

**How to cite this article**: Yapiyev, V. *et al*. Top soil physical and chemical properties in Kazakhstan across a north-south gradient. *Sci. Data*. 5:180242 doi: 10.1038/sdata.2018.242 (2018).

**Publisher’s note**: Springer Nature remains neutral with regard to jurisdictional claims in published maps and institutional affiliations.

## Supplementary Material



## Figures and Tables

**Table 1:** Description of soil sampling sites.

Sample #	Location	Site Description	Land Use/Cover	Elevation, meters (msl)	Biome	Environmental Feature	Soil type	Longitude	Latitude	Mean total precipitation, mm	Mean annual temperature, °C
1	Petropavlovsk town	shrubland near Petrapavlovsk city	shrub cover	94.133	temperate shrubland	scrubland	chernozem	69.1001	54.8933	358	1.6
2	Bishkul village	cropland	crop cover	108.423	temperate grassland	steppe soil	chernozem	69.0991	54.7481	358	1.6
3	Astrakhanka village	edge of birch forest	tree cover	139.003	temperate broadleaf forest	broadleaf forest	chernozem	69.1938	54.5430	358	1.6
4	Rublivka village	cropland	grass cover	127.015	temperate grassland	steppe soil	chernozem	69.1582	54.2703	346	1.8
5	Roshinskoe village	grassland	grass cover	127.865	temperate grassland	steppe soil	chernozem	69.1613	54.0182	346	1.8
6	Obukhovka village	grassland	grass cover	188.194	temperate grassland	steppe soil	chernozem	69.4388	53.6589	332	1.6
7	Kokshetau town	perennial grassland	shrub cover	250.348	temperate shrubland	steppe soil	chernozem	69.4724	53.2594	324	1.4
8	Kenesary village	perennial grassland	grass cover	327.493	temperate grassland	steppe soil	chernozem	69.8902	53.1069	325	1.4
9	Shuchinsk village	perennial grassland	crop cover	407.036	temperate grassland	steppe soil	chernozem	70.2038	52.8431	328	1.2
10	Makinka village	pine-birch forest	tree cover	416.058	temperate mixed forest	mixed forest	chernozem	70.5615	52.5971	330	1.4
11	Krasniy Gornyak village	perennial grassland	shrub cover	384.35	temperate grassland	steppe soil	chernozem	70.9450	52.1776	325	1.9
12	Bozaigyr	perennial grassland	grass cover	407.792	temperate grassland	steppe soil	kastanozem	71.1893	51.5024	320	2.1
40	near Astana city	perennial grassland	grass cover	363.714	temperate grassland	steppe soil	kastanozem	71.6382	51.1242	320	2.7
39	30 km to Astana city	human planted shrubs	grass cover	389	temperate shrubland	planted shrubland	kastanozem	71.9464	50.9737	311	2.7
38	Anar village	cropland	crop cover	445.994	temperate grassland	steppe soil	kastanozem	72.4297	50.6460	322	2.4
37	Oshgandy village	cropland	crop cover	555.023	temperate grassland	steppe soil	kastanozem	72.7697	50.2964	315	2.7
36	near Karaganda city	cropland	grass cover	563	temperate grassland	steppe soil	kastanozem	73.0360	49.9588	309	2.9
35	Kyzylkoy village	perennial grass	grass cover	659.52	temperate grassland	steppe soil	calcisol + solonetz	73.4302	49.2811	293	2.5
34	Aksu-Ayuly village	chestnut, rich, perennial grassland	shrub cover	729.876	temperate grassland	steppe soil	calcisol + solonetz	73.6726	48.8766	268	2.4
33	Batystau village	perennial grassland with small shrubs	shrub cover	817.191	temperate grassland	steppe soil	calcisol + solonetz	73.7275	48.4394	247	2.9
32	Zharylgap batyr	rocky grassland, small hills	grass cover	724.869	temperate grassland	steppe soil	arenosol	73.9136	48.0381	247	2.9
31	Eski Karabulak	rocky grassland	grass cover	614.742	temperate grassland	steppe soil	arenosol	74.3259	47.6743	208	4.3
30	Bektau Ata Mountains	rocky, near mountains	grass cover	597.409	temperate desert	temperate desert	arenosol + solonetz	74.7246	47.3548	197	4.9
29	Konyrat settlement	15 km from Balkhash, rocky soil	barren land	417.689	temperate desert	desert	arenosol + solonetz	74.9499	46.9384	178	6.6
28	Balkhash town	little vegetation, near Balkhash town	barren land	373	temperate desert	desert	arenosol + solonetz	74.5461	46.7488	178	6.6
27	Gulshat, Lake Balkhash	dry, little vegetation	barren land	349.686	temperate desert	desert	arenosol	74.1808	46.5502	176	6.6
26	Saryshagan, Lake Balkhash	dry, solonchak, desert shrubs	barren land	344.272	temperate desert	desert	solonchak + solonetz	73.6841	46.2546	168	7.5
25	Zhastar, Lake Balkhash	rocky, solonchak, depression	barren land	342.673	temperate desert	barren land	arenosol	73.4063	45.5847	180	8.2
24	Akerme, Lake Balkhash	dry, rocky	shrub cover	363.387	temperate desert	desert scrubland	arenosol	73.7403	45.3242	192	9.1
23	Barylbaytal, Lake Balkhash	dry, rocky	shrub cover	365.974	temperate desert	desert scrubland	arenosol	74.0153	44.9768	235	9.9
22	Mirnyi	dry, small ground unidentified shrubs	barren land	526.997	temperate desert	desert scrubland	arenosol	73.9253	44.5358	229	9.6
21	Khantau	dry, 30 m from the road, shrubs	barren land	397.082	desert shrubland	desert scrubland	arenosol	73.6996	44.1545	273	10.0
20	Kenes	crop field between tree lines	grass cover	418.163	temperate grassland	steppe soil	chernozem + solonetz	73.5786	43.9649	301	10.7
19	Birlik	floodplain valley river Shu	grass cover	457.965	temperate grassland	flood plain	regosol	73.7881	43.6658	301	10.7
18	Tasytkel	grassland	grass cover	511.611	temperate grassland	steppe soil	regosol	73.5743	43.3324	335	10.7
17	Zhdanovo	200 m from the highway	grass cover	628	temperate grassland	steppe soil	regosol	73.2451	43.0241	319	10.5
16	Merke	flood plain the Valley of Shu river	grass cover	670	temperate grassland	flood plain	arenosol	73.1852	42.9245	432	1.2
15	Kokdonen	50-70 m from the road, cropland	grass cover	751.884	montane temperate grassland	grassland	umbrisol	72.5785	42.9303	408	4.1
14	Akyrtobe	grass, cereal monoculture, mountain valley	crop cover	605.374	temperate grassland	steppe soil	umbrisol	72.0000	42.9989	424	3.0
13	5 km from Taraz town	cropland, 5 km from Taraz, perennial grass	grass cover	592.175	montane temperate grassland	grassland	umbrisol	71.5082	42.9388	404	5.7

**Table 2:** Soil carbon across the sites as total cabon (TC), total organic carbon (TOC), total inorganic carbon (TIC) in g/kg.

Sample #	TC	TOC	TIC
	g/kg	g/kg	g/kg
1	10.3	9.0	0.4
2	23.3	18.7	0.7
3	50.0	40.9	10.6
4	31.9	18.2	8.9
5	33.8	30.6	2.7
6	39.8	28.1	7.4
7	25.3	21.6	1.3
8	29.5	24.1	3.8
9	36.3	30.7	2.7
10	14.2	16.3	2.7
11	23.8	14.0	5.0
12	31.6	21.0	8.8
40	15.4	19.6	1.5
39	29.7	21.9	8.6
38	19.0	16.2	1.6
37	26.6	23.6	2.9
36	21.1	20.0	1.5
35	13.3	13.4	0.4
34	11.4	9.2	0.1
33	13.1	13.0	0.5
32	9.3	9.8	0.1
31	7.2	6.9	0.5
30	9.2	5.9	2.4
29	10.3	6.2	1.5
28	13.2	6.1	6.7
27	15.0	5.2	9.1
26	16.1	6.7	6.4
25	21.1	4.3	13.7
24	16.6	4.5	11.3
23	16.8	3.8	10.9
22	19.9	8.7	10.7
21	15.5	7.7	12.5
20	19.9	10.0	10.1
19	26.2	20.7	7.8
18	22.1	5.3	11.6
17	31.5	22.5	13.5
16	25.6	17.3	7.5
15	23.8	12.3	12.3
14	19.9	8.1	13.4
13	28.3	11.4	16.6
See also Fig. 2.			
